# Extra Uterine Fœtation

**Published:** 1882-01

**Authors:** H. Rosencrans

**Affiliations:** Elgin, Ill.


### ARTICLE X.

ELGIN, Oct. 17, 1881.

MESSRS. EDITORS:—In the October number of THE CHICAGO MEDICAL JOURNAL AND EXAMINER, just received, my attention is called to an anonymous article criticising my article in your August number, on "Extra-uterine Fœtation."

The article I allude to is on page 421, Article XIII. I hope you will let me answer him in your next JOURNAL. He asks many questions, and of course expects answers. If I did not answer them, some of the readers of your JOURNAL might think, as he says, that the article was a "rude hoax." He says, "when scrutinized by him, the narrative looks improbable on the face of it; and beyond the name of Dr. Rosencrans, bears no evidence of authenticity." Are not the names of two physicians who were present and assisted me given, and their places of residence—Dr. Sutherland, of Victoria, Texas, and Dr. Blake, of Cuero, Texas? He says, "he hopes another such operation was never performed in any other State."

This operation was performed under circumstances the most unfavorable. The patient, by long suffering, was reduced to a skeleton, praying for death. The operation was performed with only two assistants, when five would not be too many. "Article XIII" asks many questions; I will endeavor to answer as briefly as I can. First: "How was it I knew the patient was a subject for an operation, before performing the *unwarrantable* and dangerous operation of paracentesis abdominis?" Early in December, 1879, this patient called at my office, accompanied by her husband (Mr. Thos. Jordan) to consult me about her health. She thought she was pregnant, but felt differently from what she had in former pregnancies. She was certain that she was not right in her womb; stated that she was four months without her courses. My examination led me to think she was pregnant. I never saw anything more of her until I was called on 28th July following to see her at her residence. She had changed so I hardly knew her; she was emaciated, and showed unmistakable signs of great suffering. She was on her hands and knees on the bed, referring most of her suffering to the lower portion of her back. Laudanum and bromide potassium were administered. This gave her relief, sleeping most of the night. In the morning she related to me that when she was "nine months gone" she was taken with "labor pains" which lasted all one night; that up to that time she had felt motion, but none since. I think the fœtus died that night. I examined the uterus, and was sure there was not a fœtus in it. Palpation and the extreme enlargement of the abdomen satisfied me that there was fluid in the peritoneal sac or in the cyst containing the fœtus, if she was carrying one, and I believed she was. I stated to her and to her friends the necessity of an operation, explaining the nature of it. She begged to have it done, saying she would rather die under the operation than live and suffer any longer. It will be considered strange that she had not sent for a physician sooner. The only explanation is that they were poor and ignorant people, and she had depended on an old "midwife" in the neighborhood. Owing to her living in an out-of-the-way place, I advised her being carefully brought to town on a mattrass in a spring wagon. The second day after that she was brought to town. I gave her a day to rest, and then performed that "unwarrantable and dangerous operation" of paracentesis abdominis. This gave my patient immediate relief, and enabled her to eat and retain food, as well as lessening her pain. This "unwarrantable and dangerous proceeding" prepared her better to undergo the operation of the 20th.

"Why was the operation delayed so long?" As I stated in my previous article, immediately after the operation of opening the cyst with the trocar, I was enabled to more clearly make out my diagnosis. I wrote it out, and sent it to Dr. Sutherland, of Victoria, inviting him to come as soon as possible and assist me in the operation. It was a week before he came, accompanied by Dr. Blake, of Cuero, and Dr. Hopkins, of Myersville, now of Victoria. They arrived on the 9th of October. We all repaired at once to see the patient. After an examination, they agreed with me that an operation was necessary, but that in her very miserable condition, hardly a ray of hope was left. We prepared to operate next morning (10th). A violent storm of wind and rain sprang up that night, and in the morning the town was covered with water from the bay. The doctors would not remain, but left town, and half of the population of the place fled to the country for safety. (Doubtless it will be remembered that in September, 1875, a cyclone swept over that town, the waters of the bay drowning over 200 of its inhabitants, and half of the place was destroyed). This caused the delay of the operation until the 20th. Drs. Sutherland and Blake returned on the 19th. Dr. Hopkins was unavoidably delayed, which he regretted very much. At ten o'clock in the morning the operation was commenced, the patient being laid on the operating table and chloroform administered. So low was my patient at one time during the operation that it was whispered to me, "she is dead." She was allowed to recover from the influence of the chloroform, and brandy and water were fed her by the teaspoonful during the remainder of the operation. Critic says, "I cut and tore as on a cadaver;" yes, and what was a dead fœtus, a rotten placenta, and every thing belonging to it but cadaver? I was inflicting no pain while dealing with those parts; I tore where it was not safe to cut. He quotes high authority why I should not have removed the placenta. I believe that any author he quotes would have removed it. Would he have left it? With a rotten placenta left in the patient's body, and the wound closed, I certainly think she would have died.

"Where did the placenta grow?" In the most dependent part of the cyst, to the right ovary. "Why remove the right ovary?" Because it was involved in the cyst. The pregnancy was a right ovarian one.

"To what did the cyst adhere?" To the abdominal walls extensively, and to the omentum. As stated in the former article, I cut away some of the omentum. I then ligated it with catgut, and returned it in its place. "Was there no peritoneum met, and what was its condition?" The peritoneum was "met" after cutting through the abdominal walls; its condition normal. It was first taken up carefully with a tenaculum, after the bleeding was stopped, then carefully cut an inch or so, then a grooved director passed in, and a more extensive cut made. This brought to view the nauseous cyst.

Through this I could feel the fœtus. I then first passed in a medium-sized trocar, but the thick fluid running but slowly, I removed the instrument, and boldly made a cut through the cyst, turning my patient on her side—taking the precaution not to let any of the fluid run into the abdominal cavity. "How was the peritoneum dealt with in closing the wound?" It was let alone. The wound was closed without including it in the stitches. "Were the bricks put on to compress the abdominal aorta?" The bricks were put on to prevent any oozing of blood from the small vessels.

The copious discharge of putrid matter through the drainage tube was caused by portions of the cyst I had to leave, not thinking it judicious to attempt to remove all, owing to the almost lifeless condition of the patient at the time. "Is it possible for any human being to endure such cold *so long* and live?" It certainly is possible, for my patient lives. He asks "if it was necessary to practice catheterism for a month?" He had better "scrutinize" the article again. Mention in it is made of using the catheter *one week* only. "Were opiates used?" A little at first. I gave her an opiate every night for the first week after the operation, for a twofold purpose, viz., to keep the bowels from moving off, and to give her good sleep. "What is the explanation of the high pulse?" Debility. "What was the cause of the long recovery?" The drainage opening was slow in closing, and it took time to gain flesh. She gained thirty pounds while convalescing. "What was the condition of the fœtus?" It was in quite a good state of preservation, better than the umbilical cord; the latter was discolored and rotten. Counting this woman four months pregnant at the time I first saw her in December, and judging that it died the night she had "labor pains," the fœtus must have been dead about three months.

H. ROSENCRANS, M.D.

